# Supplementary material for: Pan-cancer single-cell analysis reveals the heterogeneity and plasticity of cancer-associated fibroblasts in the tumor microenvironment
Source: Nat Commun. 2022 Nov 4;13:6619. doi: 10.1038/s41467-022-34395-2 (PMC9636408; doi:10.1038/s41467-022-34395-2)
Supplement: Supplementary file 1 — Supplementary Figures [file 41467_2022_34395_MOESM1_ESM.pdf]

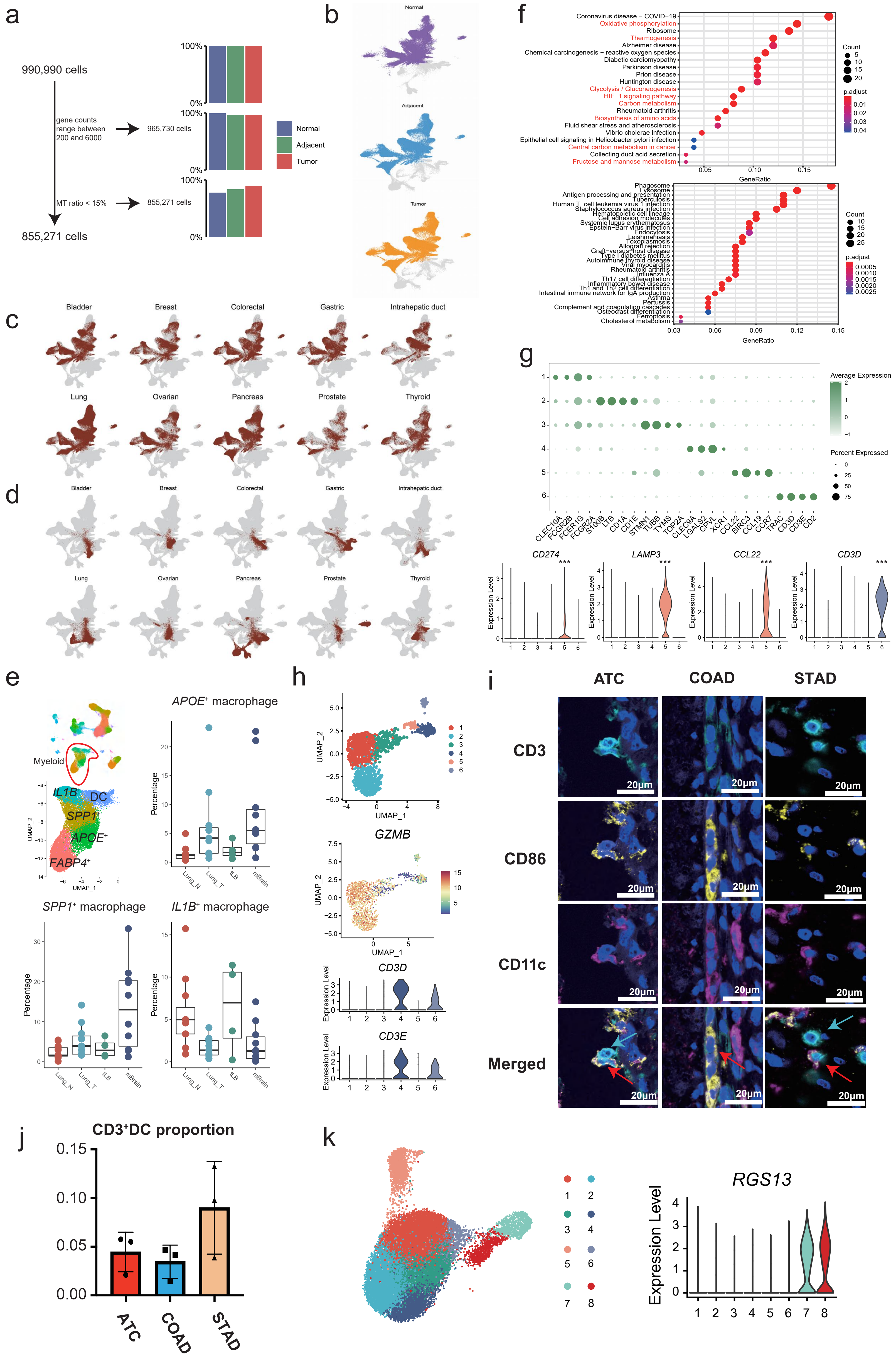
a
990,990 cells
gene counts range between 200 and 6000
965,730 cells
MT ratio < 15%
855,271 cells
855,271 cells
Normal
Adjacent
Tumor
b
Normal
Adjacent
Tumor
c
Bladder
Breast
Colorectal
Gastric
Intrahepatic duct
Lung
Ovarian
Pancreas
Prostate
Thyroid
d
e
Myeloid
IL1B+
DC
SPP1+
APOE+
FABP4+
APOE+ macrophage
SPP1+ macrophage
IL1B+ macrophage
Percentage
Lung_N
Lung_T
tLB
mBrain
f
Coronavirus disease − COVID−19
Oxidative phosphorylation
Ribosome
Thermogenesis
Alzheimer disease
Chemical carcinogenesis − reactive oxygen species
Diabetic cardiomyopathy
Parkinson disease
Prion disease
Huntington disease
Glycolysis / Gluconeogenesis
HIF−1 signaling pathway
Carbon metabolism
Rheumatoid arthritis
Biosynthesis of amino acids
Fluid shear stress and atherosclerosis
Vibrio cholerae infection
Epithelial cell signaling in Helicobacter pylori infection
Central carbon metabolism in cancer
Collecting duct acid secretion
Fructose and mannose metabolism
GeneRatio
Count
p.adjust
Phagosome
Lysosome
Antigen processing and presentation
Tuberculosis
Human T−cell leukemia virus 1 infection
Staphylococcus aureus infection
Hematopoietic cell lineage
Cell adhesion molecules
Systemic lupus erythematosus
Epstein−Barr virus infection
Endocytosis
Leishmaniasis
Toxoplasmosis
Allograft rejection
Graft−versus−host disease
Type I diabetes mellitus
Autoimmune thyroid disease
Viral myocarditis
Rheumatoid arthritis
Influenza A
Th17 cell differentiation
Inflammatory bowel disease
Th1 and Th2 cell differentiation
Intestinal immune network for IgA production
Asthma
Pertussis
Complement and coagulation cascades
Osteoclast differentiation
Ferroptosis
Cholesterol metabolism
g
Average Expression
Percent Expressed
CLEC10A
FCGR2B
FCER1G
FCGR2A
S100B
LTB
CD1A
CD1E
STMN1
TUBB
TYMS
TOP2A
CLEC9A
LGALS2
CPVL
XCR1
CCL22
BIRC3
CCL19
CCR7
TRAC
CD3D
CD3E
CD2
CD274
LAMP3
CCL22
CD3D
Expression Level
h
GZMB
CD3D
CD3E
UMAP_1
UMAP_2
i
ATC
COAD
STAD
CD3
CD86
CD11c
Merged
20μm
j
CD3+DC proportion
ATC
COAD
STAD
k
RGS13
Expression Level

Figure S1. Quality control and overall/subcluster single-cell transcriptome profile. a. Quality check pipeline of the pan-cancer single-cell profile. **b/c.** Distribution of TME cells represented by tissue types and malignant status. **d.** Distribution of epithelial cells represented by tissue types. **e.** The UMAP clustering result of myeloid cells. The alteration in the proportion of *IL1B*$^{+}$, *SPP1*$^{+}$ and *APOE*$^{+}$ macrophages along adjacent normal lung (Lung_N: n = 11), lung tumor (Lung_T: n = 11), advanced stage of tumor (tLB: n = 4), and brain metastasized (mBrain: n = 10) tissues. The box is bounded by the first and third quartile with a horizontal line at the median and whiskers extend to the maximum and minimum value. **f.** Top pathways enriched in *SPP1*$^{+}$ TAMs (upper) and *C1QC*$^{+}$ TAMs (lower) as determined by KEGG. **g.** Bubble plot of markers in each dendrite cell (DC) subcluster and the violin plot of *CD274*, *LAMP3*, *CCL22* and *CD3D,* Kruskal-Wallis two-sided test is used to test the significance of gene expression level among different DC cells clusters. ***: $p < 0.001$. **h.** Subclusters of plasmacytoid DCs (pDCs) and feature plot and violin plot of *GZMB*, *CD3D*, and *CD3E*. **i/j.** Confocal images of multiplexed immunofluorescence staining of CD3, CD11c and CD86 in anaplastic thyroid (n = 3), gastric (n = 3) and colorectal cancer (n = 3). The quantification results in each of them. The blue arrow indicates CD3^{+} T-cell, and the red arrow indicates CD3^{+} DCs (CD3^{+}CD11c^{+}CD86^{+}). Scale bar: 20 μm. Multiplexed immunofluorescence assays are performed twice on tumor samples following assay optimization. **k.** Subclustering tumor-infiltrated B-cell and violin plot with specific gene expression in subclusters. Source data are provided as a Source Data file.

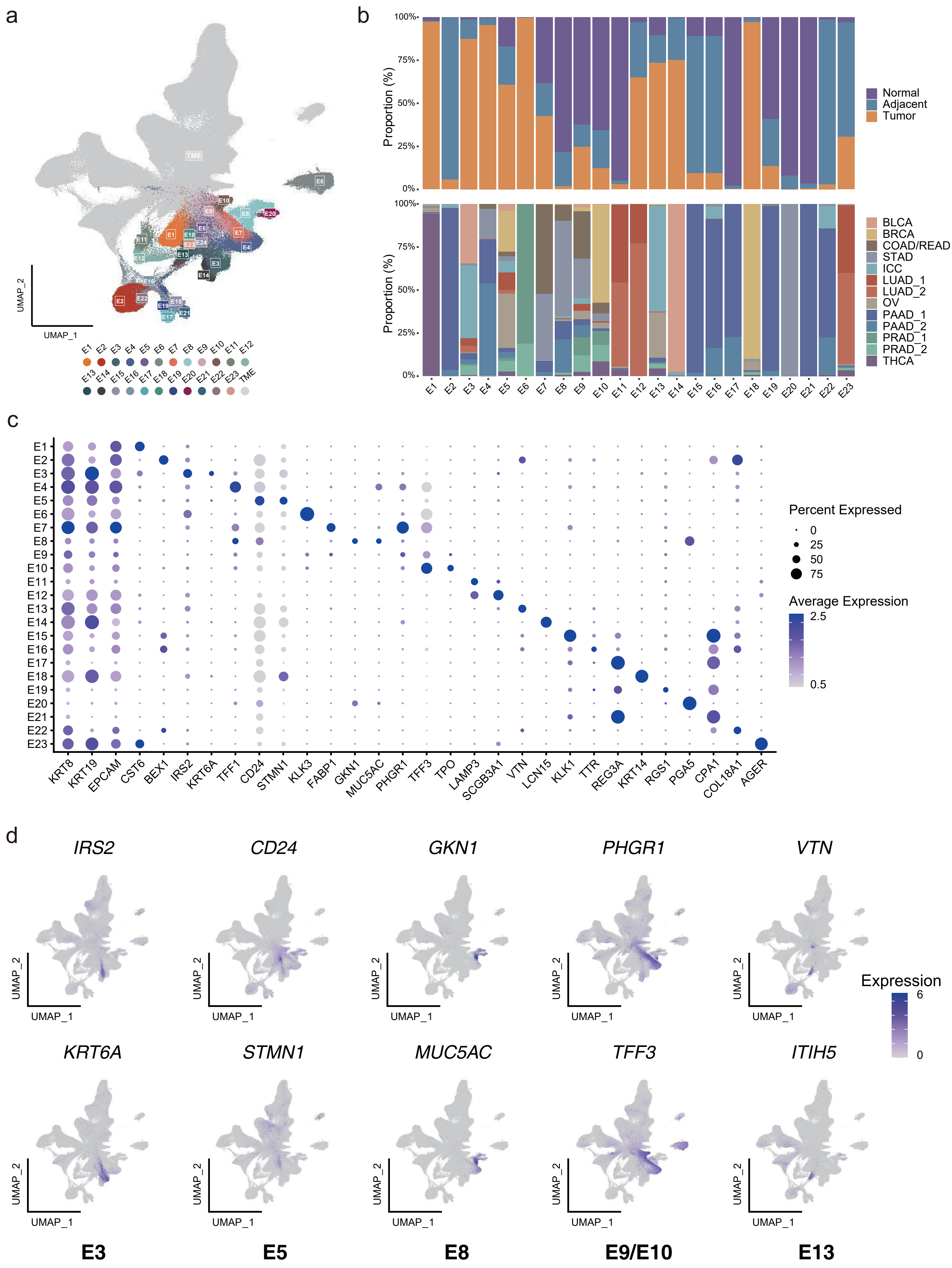
a
b
c
d
UMAP_1
UMAP_2
TME
Proportion (%)
Normal
Adjacent
Tumor
BLCA
BRCA
COAD/READ
STAD
ICC
LUAD_1
LUAD_2
OV
PAAD_1
PAAD_2
PRAD_1
PRAD_2
THCA
Percent Expressed
0
25
50
75
Average Expression
2.5
0.5
KRT8
KRT19
EPCAM
CST6
BEX1
IRS2
KRT6A
TFF1
CD24
STMN1
KLK3
FABP1
GKN1
MUC5AC
PHGR1
TFF3
TPO
LAMP3
SCGB3A1
VTN
LCN15
KLK1
TTR
REG3A
KRT14
RGS1
PGA5
CPA1
COL18A1
AGER
IRS2
CD24
GKN1
PHGR1
VTN
KRT6A
STMN1
MUC5AC
TFF3
ITIH5
Expression
6
0
E3
E5
E8
E9/E10
E13

Figure S2. Profile of epithelial clusters across different cancer types. a. The clustering of epithelial cells in pan-cancer profile. **b.** Histography of the composition proportion of different tissue types in each epithelial cluster. **c.** Bubble plot of specific expressed marker in each epithelial cluster. **d.** The feature plot of selected genes in specific epithelial clusters (E3, E5, E8, E9, E10 and E13).

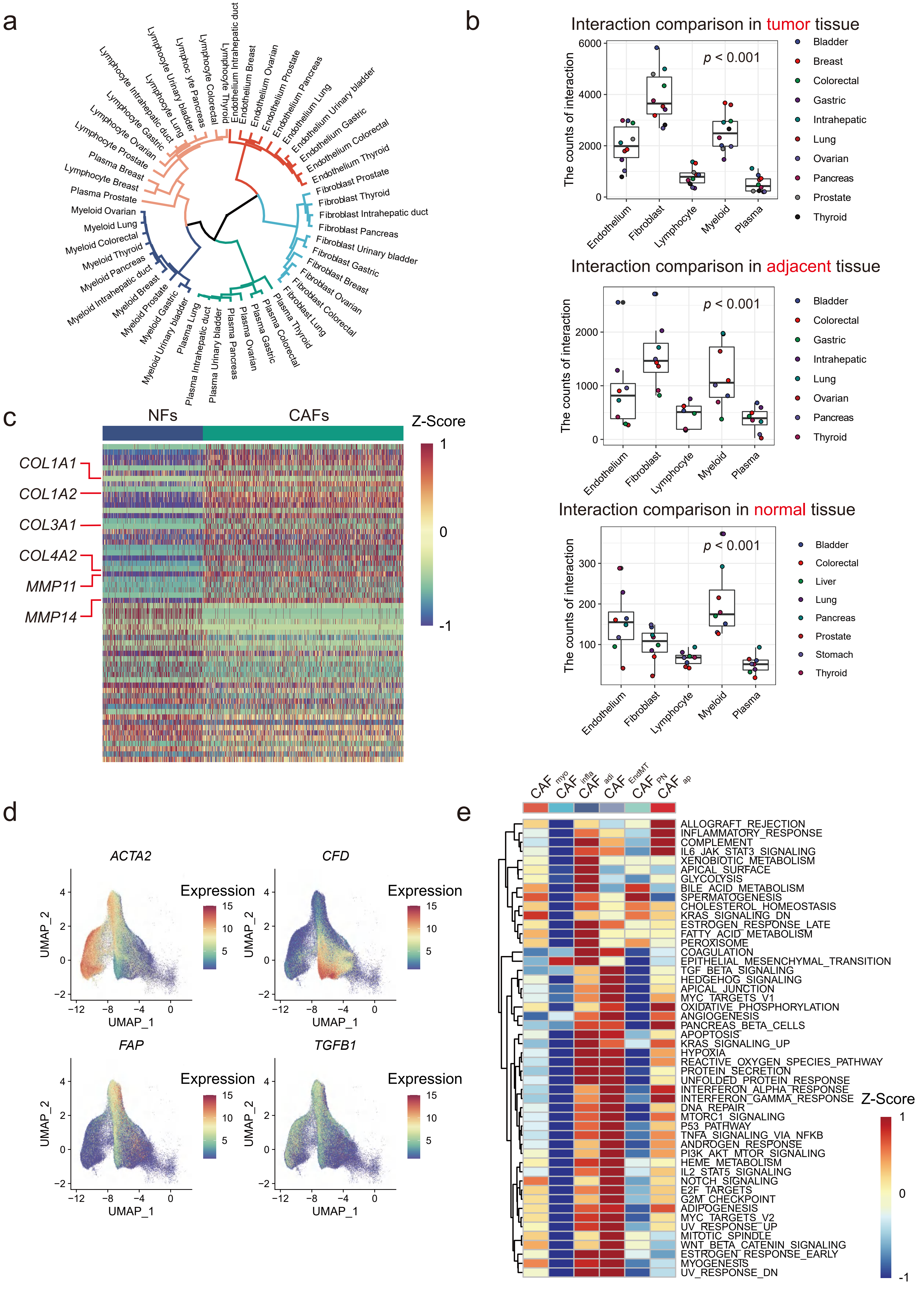
a
b
Interaction comparison in tumor tissue
The counts of interaction
6000
4000
2000
0
p < 0.001
Endothelium
Fibroblast
Lymphocyte
Myeloid
Plasma
Bladder
Breast
Colorectal
Gastric
Intrahepatic
Lung
Ovarian
Pancreas
Prostate
Thyroid
Interaction comparison in adjacent tissue
2000
1000
0
Bladder
Colorectal
Gastric
Intrahepatic
Lung
Ovarian
Pancreas
Thyroid
Interaction comparison in normal tissue
300
200
100
Bladder
Colorectal
Liver
Lung
Pancreas
Prostate
Stomach
Thyroid
Lymphocyte Intrahepatic duct
Lymphocyte Urinary bladder
Lymphoc yte Pancreas
Lymphocyte Colorectal
Lymphocyte Thyroid
Endothelium Intrahepatic duct
Endothelium Breast
Endothelium Ovarian
Endothelium Prostate
Endothelium Pancreas
Endothelium Lung
Endothelium Urinary bladder
Endothelium Gastric
Endothelium Colorectal
Endothelium Thyroid
Fibroblast Prostate
Fibroblast Thyroid
Fibroblast Intrahepatic duct
Fibroblast Pancreas
Fibroblast Urinary bladder
Fibroblast Gastric
Fibroblast Breast
Fibroblast Ovarian
Fibroblast Colorectal
Fibroblast Lung
Plasma Thyroid
Plasma Colorectal
Plasma Gastric
Plasma Ovarian
Plasma Pancreas
Plasma Urinary bladder
Plasma Intrahepatic duct
Plasma Lung
Myeloid Urinary bladder
Myeloid Gastric
Myeloid Prostate
Myeloid Breast
Myeloid Intrahepatic duct
Myeloid Pancreas
Myeloid Thyroid
Myeloid Colorectal
Myeloid Lung
Myeloid Ovarian
Plasma Prostate
Lymphocyte Breast
Plasma Breast
Lymphocyte Prostate
Lymphocyte Ovarian
Lymphocyte Gastric
Lymphocyte Lung
c
NFs
CAFs
Z-Score
1
0
-1
COL1A1
COL1A2
COL3A1
COL4A2
MMP11
MMP14
d
ACTA2
CFD
FAP
TGFB1
Expression
15
10
5
UMAP_2
UMAP_1
4
2
0
−2
−12
−8
−4
0
e
CAF myo
CAF infla
CAF adi
CAF EndMT
CAF PN
CAF ap
ALLOGRAFT_REJECTION
INFLAMMATORY_RESPONSE
COMPLEMENT
IL6_JAK_STAT3_SIGNALING
XENOBIOTIC_METABOLISM
APICAL_SURFACE
GLYCOLYSIS
BILE_ACID_METABOLISM
SPERMATOGENESIS
CHOLESTEROL_HOMEOSTASIS
KRAS_SIGNALING_DN
ESTROGEN_RESPONSE_LATE
FATTY_ACID_METABOLISM
PEROXISOME
COAGULATION
EPITHELIAL_MESENCHYMAL_TRANSITION
TGF_BETA_SIGNALING
HEDGEHOG_SIGNALING
APICAL_JUNCTION
MYC_TARGETS_V1
OXIDATIVE_PHOSPHORYLATION
ANGIOGENESIS
PANCREAS_BETA_CELLS
APOPTOSIS
KRAS_SIGNALING_UP
HYPOXIA
REACTIVE_OXYGEN_SPECIES_PATHWAY
PROTEIN_SECRETION
UNFOLDED_PROTEIN_RESPONSE
INTERFERON_ALPHA_RESPONSE
INTERFERON_GAMMA_RESPONSE
DNA_REPAIR
MTORC1_SIGNALING
P53_PATHWAY
TNFA_SIGNALING_VIA_NFKB
ANDROGEN_RESPONSE
PI3K_AKT_MTOR_SIGNALING
HEME_METABOLISM
IL2_STAT5_SIGNALING
NOTCH_SIGNALING
E2F_TARGETS
G2M_CHECKPOINT
ADIPOGENESIS
MYC_TARGETS_V2
UV_RESPONSE_UP
MITOTIC_SPINDLE
WNT_BETA_CATENIN_SIGNALING
ESTROGEN_RESPONSE_EARLY
MYOGENESIS
UV_RESPONSE_DN
Z-Score
1
0
-1

Figure S3. General characteristics of CAF. a. Hierarchical clustering of TME components in all cancer types. **b.** Tissue type-specific interaction quantification of the main TME components represented in tumor (Fibroblast: n = 10, Lymphocyte: n = 10, Myeloid: n = 10, Endothelium: n = 10, Plasma: n = 10), adjacent (Fibroblast: n = 8, Lymphocyte: n = 8, Myeloid: n = 8, Endothelium: n = 8, Plasma: n = 8), and normal tissues (Fibroblast: n = 7, Lymphocyte: n = 7, Myeloid: n = 7, Endothelium: n = 7, Plasma: n = 7). The box is bounded by the first and third quartile with a horizontal line at the median and whiskers extend to the maximum and minimum value, Anova two-sided test is used to test the significance of counts of interaction among different cell type categories. Tumor *p*-value is 3.93×10^{-14}. Adjacent *p*-value is 2.38×10^{-4}, Normal tissue *p*-value is 8.19×10^{-5}. **c.** Differentially expressed genes between cancer-associated fibroblasts (CAFs) and normal fibroblasts (NFs). **d.** Feature plots of *ACTA2*, *ACTG2*, *FAP*, and *TGFB1* in the fibroblast component of the TME. **e.** Gene ontology enrichment of all components of CAFs. Source data are provided as a Source Data file.

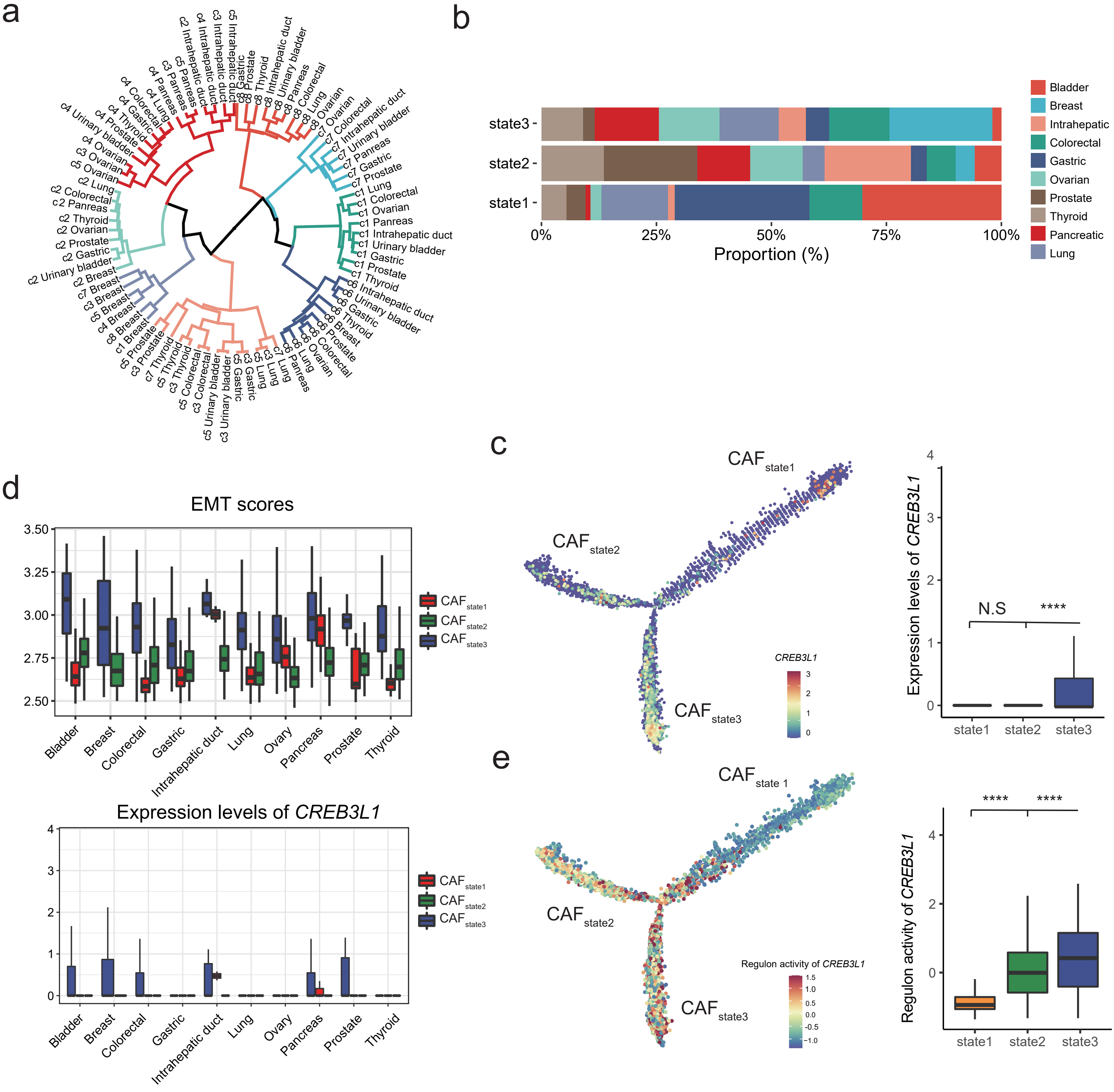
a
c4 Urinary bladder
c4 Prostate
c4 Thyroid
c4 Gastric
c4 Colorectal
c4 Lung
c4 Panreas
c3 Panreas
c5 Panreas
c2 Intrahepatic duct
c4 Intrahepatic duct
c3 Intrahepatic duct
c5 Intrahepatic duct
c8 Gastric
c8 Prostate
c8 Thyroid
c8 Intrahepatic duct
c8 Urinary bladder
c8 Panreas
c8 Colorectal
c8 Lung
c8 Ovarian
c7 Ovarian
c7 Colorectal
c7 Intrahepatic duct
c7 Urinary bladder
c7 Panreas
c7 Gastric
c7 Prostate
c1 Lung
c1 Colorectal
c1 Ovarian
c1 Panreas
c1 Intrahepatic duct
c1 Urinary bladder
c1 Gastric
c1 Prostate
c1 Thyroid
c6 Intrahepatic duct
c6 Urinary bladder
c6 Gastric
c6 Thyroid
c6 Breast
c6 Prostate
c6 Colorectal
c6 Ovarian
c6 Lung
c6 Panreas
c7 Lung
c3 Lung
c5 Lung
c3 Gastric
c5 Gastric
c3 Urinary bladder
c5 Urinary bladder
c3 Colorectal
c5 Colorectal
c3 Thyroid
c5 Thyroid
c7 Thyroid
c3 Prostate
c5 Prostate
c1 Breast
c8 Breast
c4 Breast
c5 Breast
c3 Breast
c7 Breast
c2 Breast
c2 Urinary bladder
c2 Gastric
c2 Prostate
c2 Ovarian
c2 Thyroid
c2 Panreas
c2 Colorectal
c2 Lung
c5 Ovarian
c3 Ovarian
c4 Ovarian
b
state3
state2
state1
0%
25%
50%
75%
100%
Proportion (%)
Bladder
Breast
Intrahepatic
Colorectal
Gastric
Ovarian
Prostate
Thyroid
Pancreatic
Lung
c
CAF state1
CAF state2
CAF state3
CREB3L1
3
2
1
0
4
Expression levels of CREB3L1
N.S
****
state1
state2
state3
d
EMT scores
3.50
3.25
3.00
2.75
2.50
CAF state1
CAF state2
CAF state3
Bladder
Breast
Colorectal
Gastric
Intrahepatic duct
Lung
Ovary
Pancreas
Prostate
Thyroid
Expression levels of CREB3L1
4
3
2
1
0
e
CAF state 1
CAF state2
CAF state3
Regulon activity of CREB3L1
1.5
1.0
0.5
0.0
−0.5
−1.0
****
****
Regulon activity of CREB3L1
4
2
0
state1
state2
state3

Figure S4. Characteristics of three CAF states. a. Hierarchical clustering of fibroblasts in all cancer types. **b.** The constitution ratio of each cancer type in each state. **c.** Left: *CREB3L1* expression was enriched along the evolutionary trajectory of CAFs. Right: comparison of *CREB3L1* expression among the three CAF states, state1: n = 2133, state2: n = 2476, state3: n = 2891, The box is bounded by the first and third quartile with a horizontal line at the median and whiskers extend to the maximum and minimum value. Mann–Whitney two-sided test is used to test the significance of expression level of *CREB3L1* between CAF states. ****: $p < 0.0001$. **d.** Comparison of the EMT score and *CREB3L1* expression among the three states of CAFs in each tissue type, Bladder: n = 1448, Breast: n = 454, Colorectal: n = 706, Gastric: n = 821, Intrahepatic duct: n = 59, Lung: n = 668, Ovary: n = 1262, Pancreas: n = 1659, Prostate: n = 168, Thyroid: n = 248. **e.** Left: *CREB3L1* downstream target set enriched along the evolutionary trajectory of CAFs. Right: comparison of the downstream target set of *CREB3L1* among the three CAF states, state1: n = 2133, state2: n = 2476, state3: n = 2891. The box is bounded by the first and third quartile with a horizontal line at the median and whiskers extend to the maximum and minimum value. Mann–Whitney two-sided test is used to test the significance of regulon activity of *CREB3L1* between CAF states. ****: $p < 0.0001$. Source data are provided as a Source Data file.

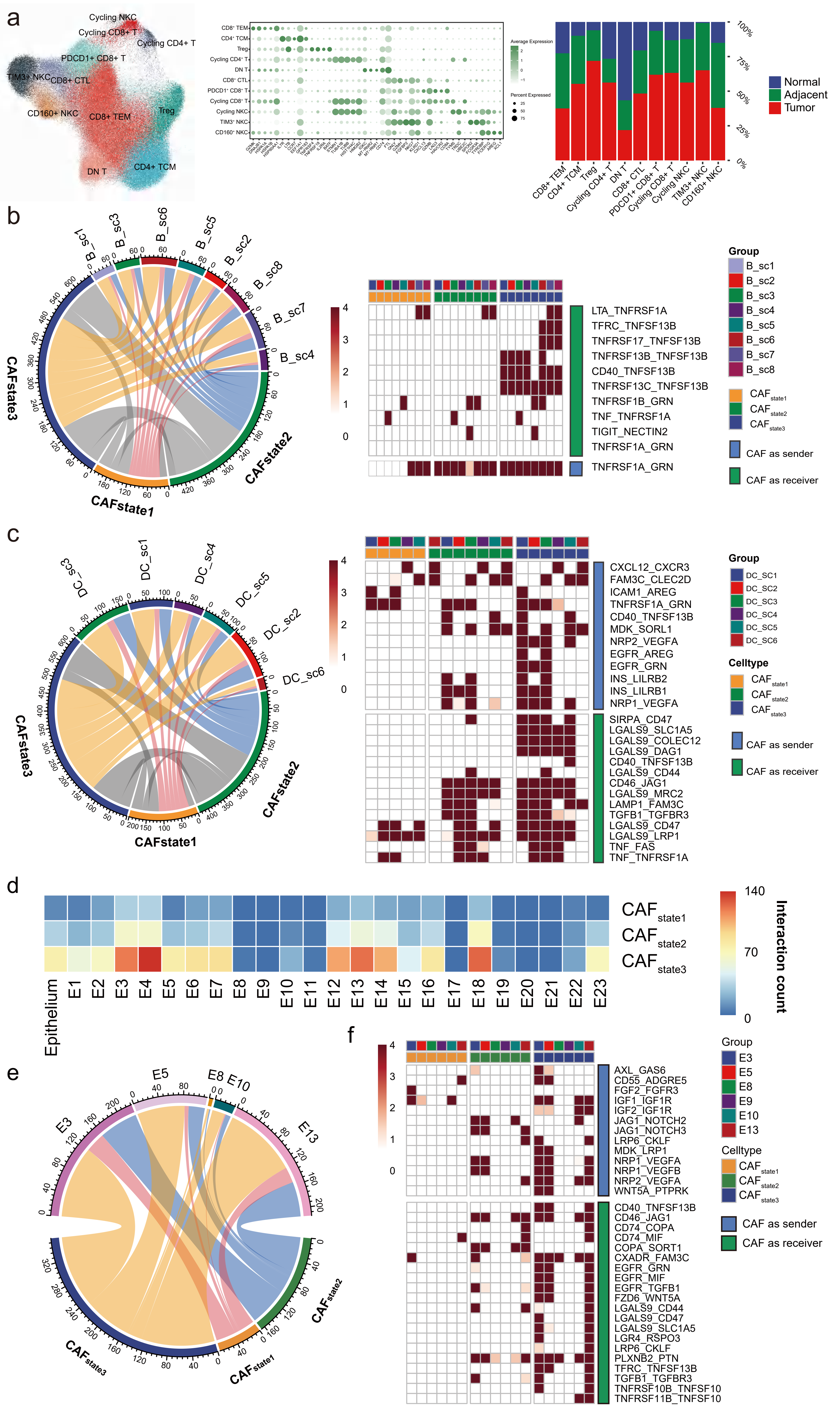
a
Cycling NKC
Cycling CD8+ T
Cycling CD4+ T
PDCD1+ CD8+ T
TIM3+ NKC
CD8+ CTL
CD160+ NKC
CD8+ TEM
Treg
DN T
CD4+ TCM
Average Expression
Percent Expressed
Normal
Adjacent
Tumor
100%
75%
50%
25%
0%
CD8+ TEM
CD4+ TCM
Treg
Cycling CD4+ T
DN T
CD8+ CTL
PDCD1+ CD8+ T
Cycling CD8+ T
Cycling NKC
TIM3+ NKC
CD160+ NKC
b
B_sc1
B_sc3
B_sc6
B_sc5
B_sc2
B_sc8
B_sc7
B_sc4
CAFstate3
CAFstate2
CAFstate1
LTA_TNFRSF1A
TFRC_TNFSF13B
TNFRSF17_TNFSF13B
TNFRSF13B_TNFSF13B
CD40_TNFSF13B
TNFRSF13C_TNFSF13B
TNFRSF1B_GRN
TNF_TNFRSF1A
TIGIT_NECTIN2
TNFRSF1A_GRN
TNFRSF1A_GRN
Group
B_sc1
B_sc2
B_sc3
B_sc4
B_sc5
B_sc6
B_sc7
B_sc8
CAFstate1
CAFstate2
CAFstate3
CAF as sender
CAF as receiver
c
DC_sc3
DC_sc1
DC_sc4
DC_sc5
DC_sc2
DC_sc6
CAFstate3
CAFstate2
CAFstate1
CXCL12_CXCR3
FAM3C_CLEC2D
ICAM1_AREG
TNFRSF1A_GRN
CD40_TNFSF13B
MDK_SORL1
NRP2_VEGFA
EGFR_AREG
EGFR_GRN
INS_LILRB2
INS_LILRB1
NRP1_VEGFA
SIRPA_CD47
LGALS9_SLC1A5
LGALS9_COLEC12
LGALS9_DAG1
CD40_TNFSF13B
LGALS9_CD44
CD46_JAG1
LGALS9_MRC2
LAMP1_FAM3C
TGFB1_TGFBR3
LGALS9_CD47
LGALS9_LRP1
TNF_FAS
TNF_TNFRSF1A
Group
DC_SC1
DC_SC2
DC_SC3
DC_SC4
DC_SC5
DC_SC6
Celltype
CAFstate1
CAFstate2
CAFstate3
CAF as sender
CAF as receiver
d
CAFstate1
CAFstate2
CAFstate3
Epithelium
E1 E2 E3 E4 E5 E6 E7 E8 E9 E10 E11 E12 E13 E14 E15 E16 E17 E18 E19 E20 E21 E22 E23
140
70
0
Interaction count
e
E3
E5
E8
E10
E13
CAFstate2
CAFstate1
CAFstate3
f
AXL_GAS6
CD55_ADGRE5
FGF2_FGFR3
IGF1_IGF1R
IGF2_IGF1R
JAG1_NOTCH2
JAG1_NOTCH3
LRP6_CKLF
MDK_LRP1
NRP1_VEGFA
NRP1_VEGFB
NRP2_VEGFA
WNT5A_PTPRK
CD40_TNFSF13B
CD46_JAG1
CD74_COPA
CD74_MIF
COPA_SORT1
CXADR_FAM3C
EGFR_GRN
EGFR_MIF
EGFR_TGFB1
FZD6_WNT5A
LGALS9_CD44
LGALS9_CD47
LGALS9_SLC1A5
LGR4_RSPO3
LRP6_CKLF
PLXNB2_PTN
TFRC_TNFSF13B
TGFB1_TGFBR3
TNFRSF10B_TNFSF10
TNFRSF11B_TNFSF10
Group
E3
E5
E8
E9
E10
E13
Celltype
CAFstate1
CAFstate2
CAFstate3
CAF as sender
CAF as receiver

Figure S5. Interactions of CAFs with other TME components. a. Left: Clusters of NK/T cells in pan-cancer analysis. Right: bubble plot showing the specific marker genes in each cluster and the histography of the composition proportion of each type of NK/T cell. **b.** Predicted and detailed interactions between the three CAF states and each B-cell subcluster. **c.** Predicted and detailed interaction between the three CAF states with DCs and pDCs. **d.** the heatmap of interaction between each cluster of epithelial cells and CAF state1-3. **e/f.** Predicted and detailed interactions between the three CAF states and each epithelial cluster.

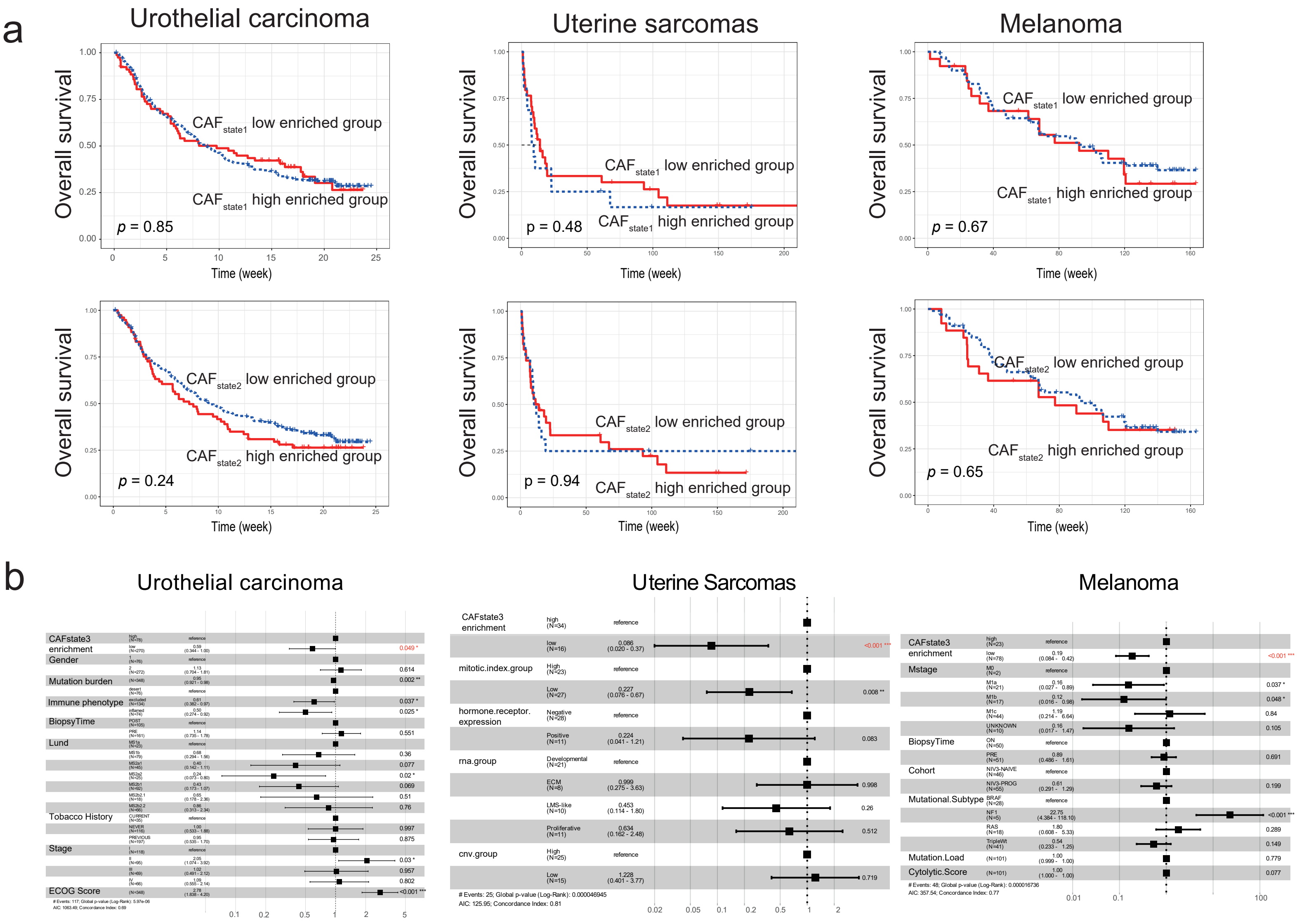

a
Urothelial carcinoma
Uterine sarcomas
Melanoma
Overall survival
Time (week)
CAFstate1 low enriched group
CAFstate1 high enriched group
CAFstate2 low enriched group
CAFstate2 high enriched group
p = 0.85
p = 0.48
p = 0.67
p = 0.24
p = 0.94
p = 0.65
b
Urothelial carcinoma
Uterine Sarcomas
Melanoma
CAFstate3 enrichment
Gender
Mutation burden
Immune phenotype
BiopsyTime
Lund
Tobacco History
Stage
ECOG Score
mitotic.index.group
hormone.receptor. expression
rna.group
cnv.group
Mstage
Cohort
Mutational.Subtype
Mutation.Load
Cytolytic.Score

Figure S6. Association of CAF states with immunotherapy survival outcomes. a. Estimation of the prognostic value of the $CAF_{state1/2}$ signature score in three immunotherapy cohorts (urothelial carcinoma, uterine sarcoma, and melanoma), Kaplan–Meier curves for overall survival in all patients according to the number of positive ligands. *p*-values for all survival analyses have been calculated using the log-rank test. **b.** The forest plot of multivariate adjustment results in three cohorts, Error bars represent 95% confidence intervals.

.

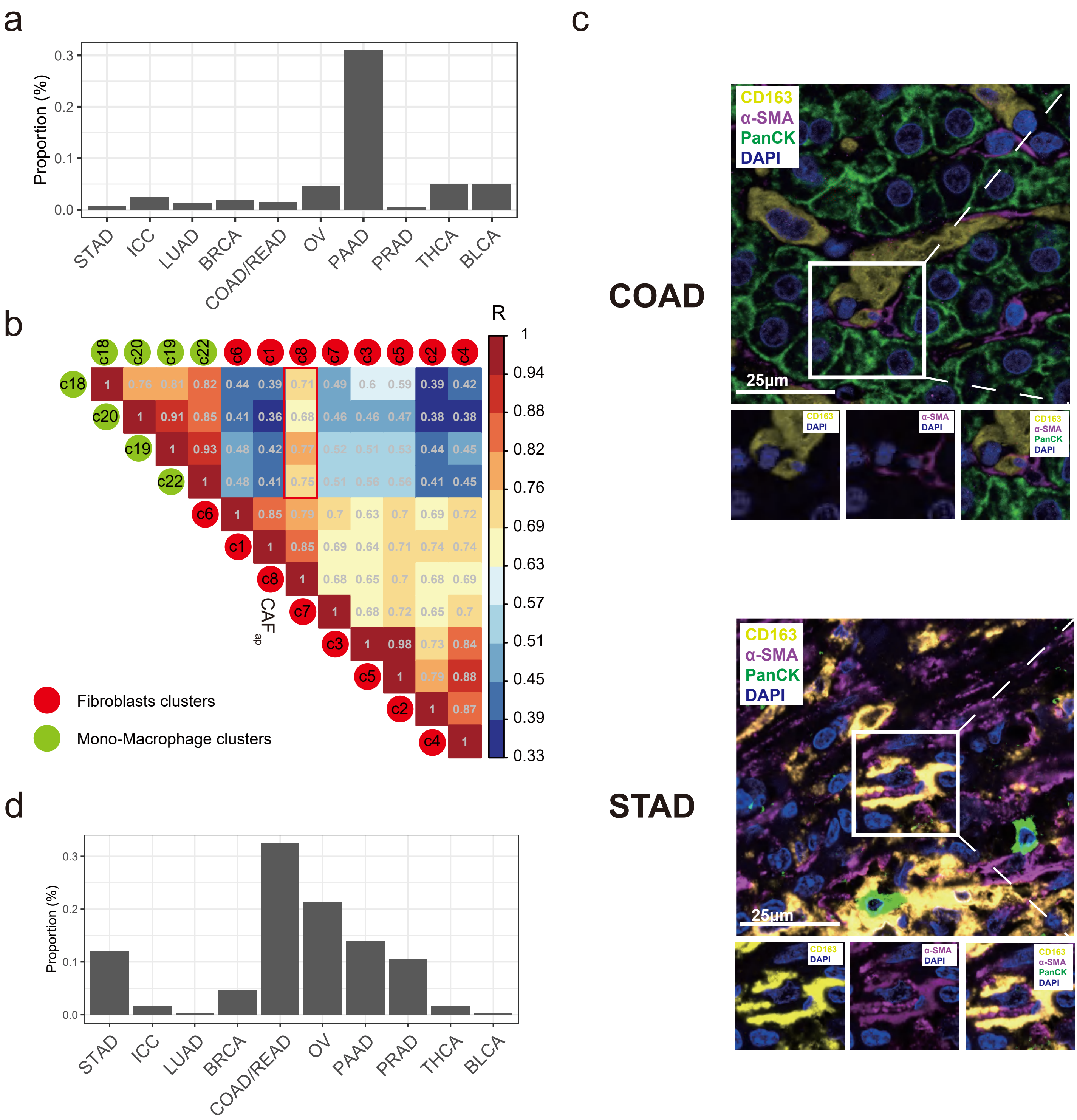
a
Proportion (%)
0.3
0.2
0.1
0.0
STAD
ICC
LUAD
BRCA
COAD/READ
OV
PAAD
PRAD
THCA
BLCA
b
R
c18 c20 c19 c22 c6 c1 c8 c7 c3 c5 c2 c4
c18 1 0.76 0.81 0.82 0.44 0.39 0.71 0.49 0.6 0.59 0.39 0.42
c20 1 0.91 0.85 0.41 0.36 0.68 0.46 0.46 0.47 0.38 0.38
c19 1 0.93 0.48 0.42 0.77 0.52 0.51 0.53 0.44 0.45
c22 1 0.48 0.41 0.75 0.51 0.56 0.56 0.41 0.45
c6 1 0.85 0.79 0.7 0.63 0.7 0.69 0.72
c1 1 0.85 0.69 0.64 0.71 0.74 0.74
c8 1 0.68 0.65 0.7 0.68 0.69
CAF ap
c7 1 0.68 0.72 0.65 0.7
c3 1 0.98 0.73 0.84
c5 1 0.79 0.88
c2 1 0.87
c4 1
1
0.94
0.88
0.82
0.76
0.69
0.63
0.57
0.51
0.45
0.39
0.33
Fibroblasts clusters
Mono-Macrophage clusters
c
COAD
CD163
α-SMA
PanCK
DAPI
25μm
STAD
CD163
α-SMA
PanCK
DAPI
25μm
d
Proportion (%)
0.3
0.2
0.1
0.0
STAD
ICC
LUAD
BRCA
COAD/READ
OV
PAAD
PRAD
THCA
BLCA

Figure S7. Characteristics of CAF_{ap} and CAF_{PN}. a. The absolute proportion of CAF_{ap} to all fibroblasts in each cancer type. **b.** Genetic similarity heatmap of fibroblast-related clusters and mono-macrophage-related clusters in the TME. **c.** Confocal images of multiplexed immunofluorescence staining of PanCK, α-SMA, and CD163 in colorectal and gastric cancer tissues. Multiplexed immunofluorescence assays are performed twice on tumor samples following assay optimization. **d.** The absolute proportion of CAF_{PN} to all fibroblasts in each cancer type.

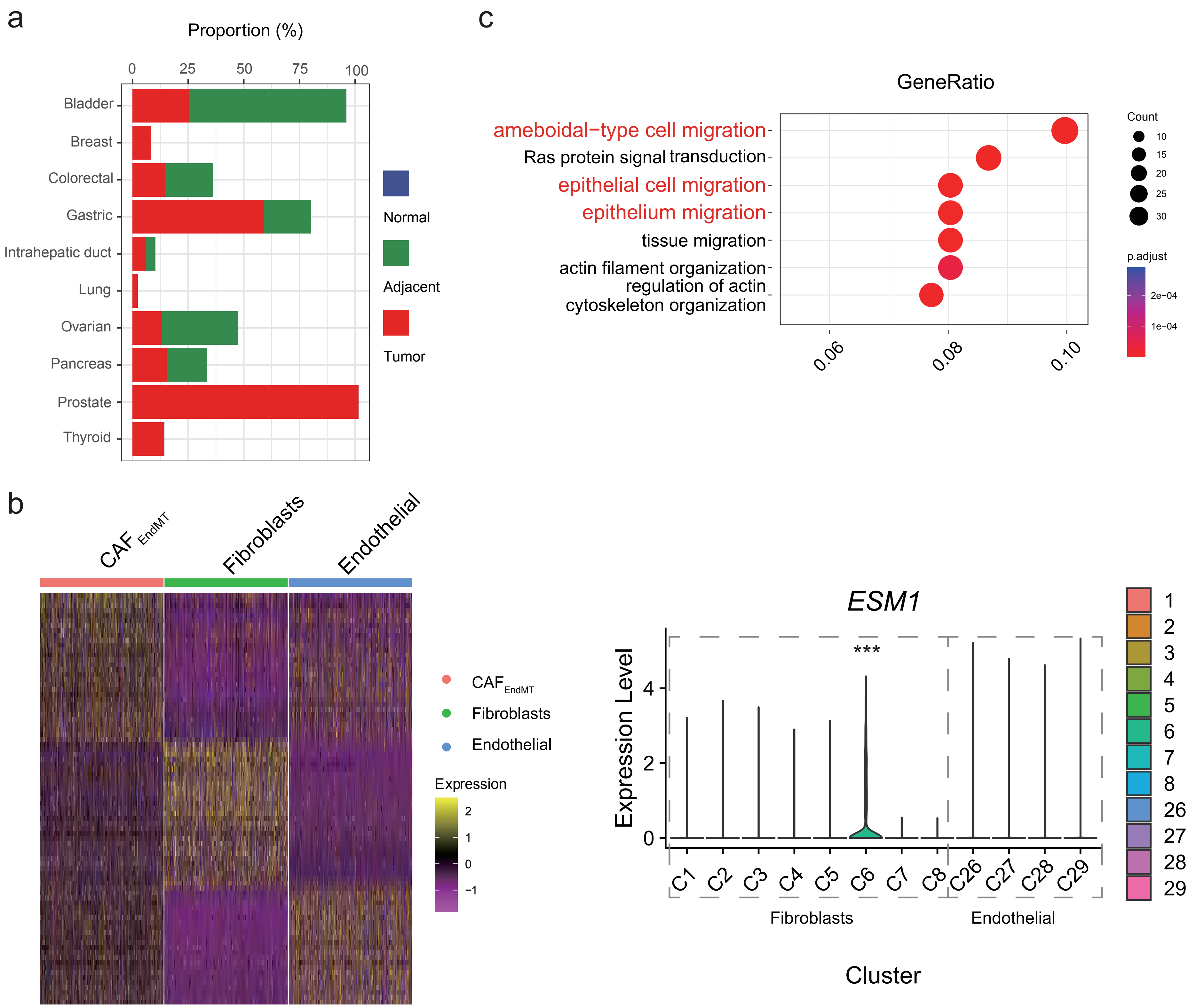
a
Proportion (%)
0
25
50
75
100
Bladder
Breast
Colorectal
Gastric
Intrahepatic duct
Lung
Ovarian
Pancreas
Prostate
Thyroid
Normal
Adjacent
Tumor
c
GeneRatio
ameboidal−type cell migration
Ras protein signal transduction
epithelial cell migration
epithelium migration
tissue migration
actin filament organization
regulation of actin cytoskeleton organization
0.06
0.08
0.10
Count
10
15
20
25
30
p.adjust
2e−04
1e−04
b
CAF EndMT
Fibroblasts
Endothelial
CAF EndMT
Fibroblasts
Endothelial
Expression
2
1
0
−1
ESM1
***
Expression Level
0
2
4
C1
C2
C3
C4
C5
C6
C7
C8
C26
C27
C28
C29
Fibroblasts
Endothelial
Cluster
1
2
3
4
5
6
7
8
26
27
28
29

Figure S8. Characteristics of CAF_{EndMT}. a. Histography of the proportion of CAF_{EndMT} in each type of cancer and cell orgins. **b.** The heatmap of differentially expressed genes in CAF_{EndMT}, all other fibroblasts and endothelial cells. Violin plot of *ESM1* in fibroblast and endothelial clusters. Kruskal-Wallis two-sided test is used to test the significance of gene expression level among different fibroblast and endothelium clusters. ***: $p < 0.001$. **c.** The KEGG pathway enrichment plot of CAF_{EndMT}. FDR value is calculated by R package "clusterProfiler".

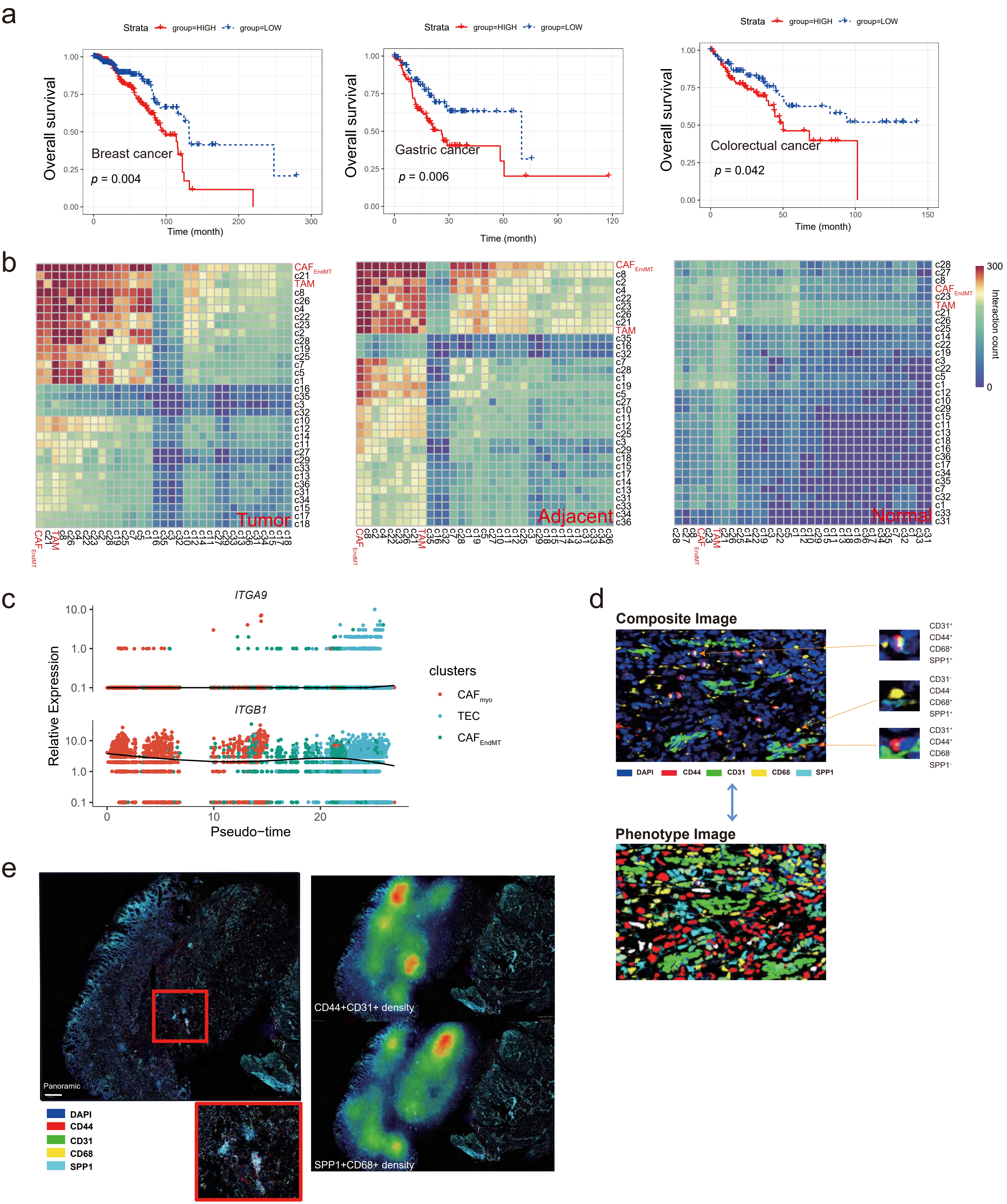
a
Strata group=HIGH group=LOW
Overall survival
Time (month)
Breast cancer
p = 0.004
Gastric cancer
p = 0.006
Colorectual cancer
p = 0.042
b
Tumor
Adjacent
Normal
CAF EndMT
TAM
Interaction count
300
0
c
ITGA9
ITGB1
Relative Expression
Pseudo−time
clusters
CAF myo
TEC
CAF EndMT
d
Composite Image
DAPI
CD44
CD31
CD68
SPP1
CD31+ CD44+ CD68+ SPP1+
CD31- CD44- CD68+ SPP1+
CD31+ CD44+ CD68- SPP1-
Phenotype Image
e
Panoramic
DAPI
CD44
CD31
CD68
SPP1
CD44+CD31+ density
SPP1+CD68+ density

Figure S9. Communications of CAF_{EndMT} with other TME components. a. Estimation of the prognostic value of the CAF_{EndMT} signature in colorectal, gastric and breast cancer in terms of overall survival, Kaplan–Meier curves for overall survival in all patients according to the number of positive ligands. *p*-values for all survival analyses have been calculated using the log-rank test. **b.** The detailed interaction heatmap among TME components in normal, adjacent, and tumor tissues. **c.** Dynamic alteration of *ITGA9* and *ITGB1* along the EndMT trajectory. **d.** Illustration of the phenotype map and density map. **e.** An example of a defective region where tissues were dropped or overturned on a slide is highlighted in a red square. Scale bar: 500 μm Multiplexed immunofluorescence assays are performed twice on tumor samples following assay optimization.

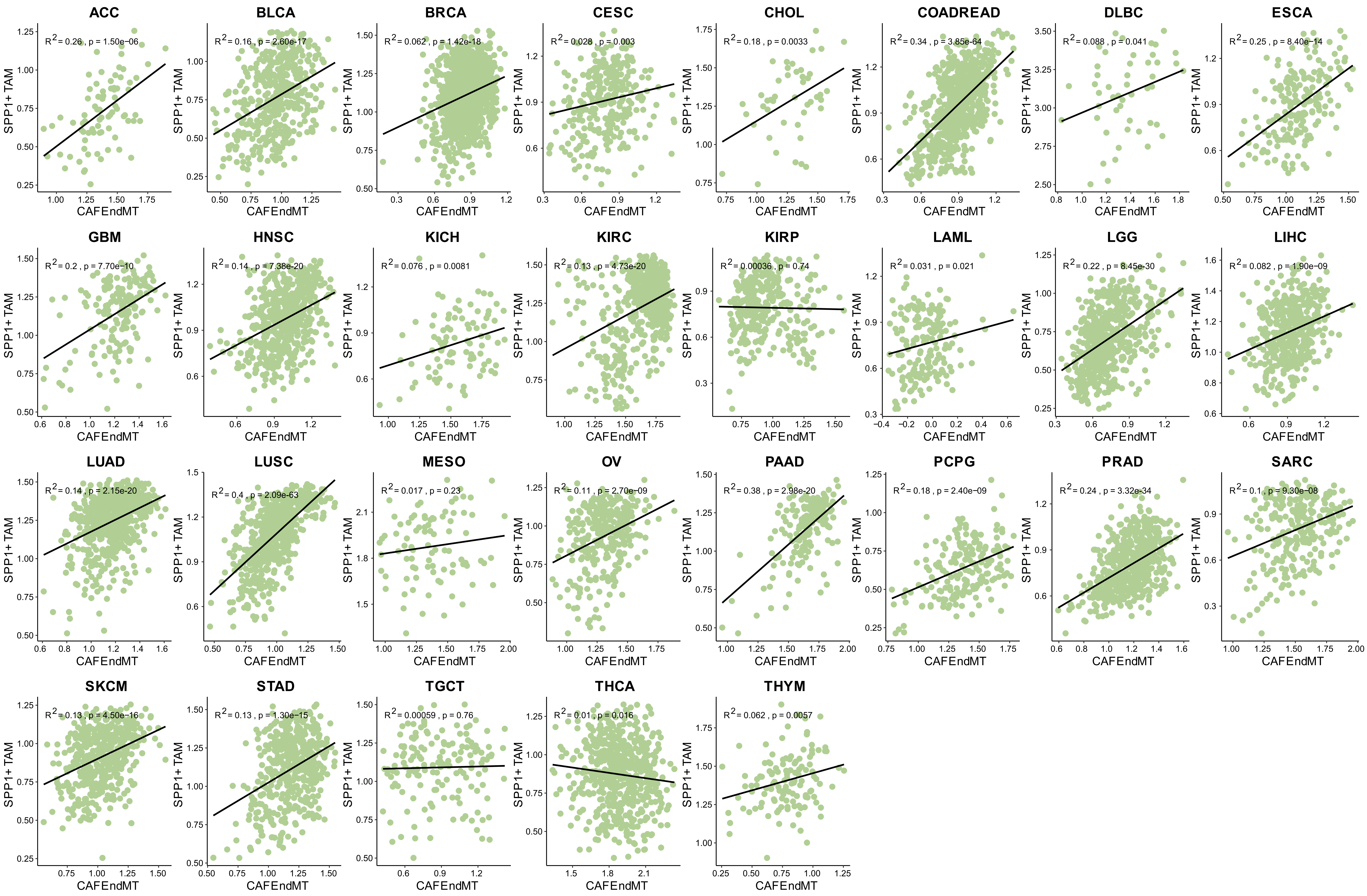
ACC
R2 = 0.26 , p = 1.50e−06
BLCA
R2 = 0.16 , p = 2.60e−17
BRCA
R2 = 0.062 , p = 1.42e−18
CESC
R2 = 0.028 , p = 0.003
CHOL
R2 = 0.18 , p = 0.0033
COADREAD
R2 = 0.34 , p = 3.85e−64
DLBC
R2 = 0.088 , p = 0.041
ESCA
R2 = 0.25 , p = 8.40e−14
GBM
R2 = 0.2 , p = 7.70e−10
HNSC
R2 = 0.14 , p = 7.38e−20
KICH
R2 = 0.076 , p = 0.0081
KIRC
R2 = 0.13 , p = 4.73e−20
KIRP
R2 = 0.00036 , p = 0.74
LAML
R2 = 0.031 , p = 0.021
LGG
R2 = 0.22 , p = 8.45e−30
LIHC
R2 = 0.082 , p = 1.90e−09
LUAD
R2 = 0.14 , p = 2.15e−20
LUSC
R2 = 0.4 , p = 2.09e−63
MESO
R2 = 0.017 , p = 0.23
OV
R2 = 0.11 , p = 2.70e−09
PAAD
R2 = 0.38 , p = 2.98e−20
PCPG
R2 = 0.18 , p = 2.40e−09
PRAD
R2 = 0.24 , p = 3.32e−34
SARC
R2 = 0.1 , p = 9.30e−08
SKCM
R2 = 0.13 , p = 4.50e−16
STAD
R2 = 0.13 , p = 1.30e−15
TGCT
R2 = 0.00059 , p = 0.76
THCA
R2 = 0.01 , p = 0.016
THYM
R2 = 0.062 , p = 0.0057
SPP1+ TAM
CAFEndMT

Figure S10. Correlation of CAF_{EndMT} with *SPP1*$^{+}$ TAM across different cancer types. Correlation of the CAF_{EndMT} and *SPP1*$^{+}$TAM signature enrichment scores in various cancer types in TCGA datasets, R represents Pearson's correlation and its coefficient of determination. Significantly positive correlations were detected in most cancer types (25 of 28).
