# Supplementary material for: Pan-cancer single-cell analysis reveals the heterogeneity and plasticity of cancer-associated fibroblasts in the tumor microenvironment
Source: Nat Commun. 2022 Nov 4;13:6619. doi: 10.1038/s41467-022-34395-2 (PMC9636408; doi:10.1038/s41467-022-34395-2)
Supplement: Supplementary file 4 — Reporting Summary [file 41467_2022_34395_MOESM4_ESM.pdf]

nature portfolio

Corresponding author(s): Heng Xu

Last updated by author(s): Mar 15, 2022

Reporting Summary

Nature Portfolio wishes to improve the reproducibility of the work that we publish. This form provides structure for consistency and transparency in reporting. For further information on Nature Portfolio policies, see our Editorial Policies and the Editorial Policy Checklist.

Statistics

For all statistical analyses, confirm that the following items are present in the figure legend, table legend, main text, or Methods section.

n/a	Confirmed	
☐	☒	The exact sample size (*n*) for each experimental group/condition, given as a discrete number and unit of measurement
☐	☒	A statement on whether measurements were taken from distinct samples or whether the same sample was measured repeatedly
☐	☒	The statistical test(s) used AND whether they are one- or two-sided<br>*Only common tests should be described solely by name; describe more complex techniques in the Methods section.*
☒	☐	A description of all covariates tested
☒	☐	A description of any assumptions or corrections, such as tests of normality and adjustment for multiple comparisons
☐	☒	A full description of the statistical parameters including central tendency (e.g. means) or other basic estimates (e.g. regression coefficient) AND variation (e.g. standard deviation) or associated estimates of uncertainty (e.g. confidence intervals)
☐	☒	For null hypothesis testing, the test statistic (e.g. *F*, *t*, *r*) with confidence intervals, effect sizes, degrees of freedom and *P* value noted<br>*Give P values as exact values whenever suitable.*
☒	☐	For Bayesian analysis, information on the choice of priors and Markov chain Monte Carlo settings
☒	☐	For hierarchical and complex designs, identification of the appropriate level for tests and full reporting of outcomes
☐	☒	Estimates of effect sizes (e.g. Cohen's *d*, Pearson's *r*), indicating how they were calculated

Our web collection on statistics for biologists contains articles on many of the points above.

Software and code

Policy information about availability of computer code

Data collection: no software was used for data collection

Data analysis:

open source tools:
Cell Ranger v6.1.2
Seurat v4.0.4
SeuratWrappers v0.3.0
circlize v0.4.14
infercnv v1.10.1
monocle v2.22.0
ggridges v0.5.3
clusterProfiler v3.0.4
escape v1.4.0
GSVA v1.14.1
SCENIC v1.2.4
psych v2.2.5
corrplot v0.92
survival v2.42-3
survminer v0.4.9
Space Ranger v1.3.1
NicheNet v1.0.0
cellphoneDB v3.0
fastMNN v1.10.0
pheatmap v1.0.12

Scrublet v0.1

Code Availability: The bioinformatic analysis code has been uploaded into the github (https://github.com/Xiaxy-XuLab/PanCAF, DOI: 10.5281/zenodo.7095147)

For manuscripts utilizing custom algorithms or software that are central to the research but not yet described in published literature, software must be made available to editors and reviewers. We strongly encourage code deposition in a community repository (e.g. GitHub). See the Nature Portfolio guidelines for submitting code & software for further information.

Data

Policy information about availability of data

All manuscripts must include a data availability statement. This statement should provide the following information, where applicable:

- Accession codes, unique identifiers, or web links for publicly available datasets
- A description of any restrictions on data availability
- For clinical datasets or third party data, please ensure that the statement adheres to our policy

All the expression data can be obtained from the Gene Expression Omnibus, and the selected studies are listed in Supplementary Data 1. Analysis and visualization of the scRNA-seq datasets in this study can also be performed at https://gist-fgl.github.io/sc-caf-atlas/. Additionally, the data are also available from the corresponding author once needed. Additionally, the integrated single-cell RNA sequencing matrix data that support the findings of this study are deposited in Gene expression Omnibus (accession No. GSE210347). Previously published scRNA-seq data reanalyzed here are available under accession codes GSE134355 (Normal data by Han et al.), GSE141445 (Prostate cancer data by Chen et al.), E-MTAB-8107 (Ovary/Breast/Colorectum cancer data by Qian et al.), GSE157703 (Prostate cancer data by Ma et al.), GSE131907 (Lung cancer data by Kim et al.), GSE138709 (Intrahepatic cholangiocarcinoma data by Zhang et al.), E-MTAB-6149, E-MTAB-6653 (Lung carcinomas data by Qian et al.), CRA001160 (PDAC data by Peng et al.), GSE154778 (PDAC data by Lin et al.), HRA000212 (Bladder cancer data by Chen et al.), HRA000686 (Thyroid cancer data by Luo et al.). The gastric cancer data by Sathe et al. were downloaded from https://dna-discovery.stanford.edu/research/datasets/. The raw sequencing data for the spatial transcriptome from this study have been deposited in the Genome Sequence Archive in BIG Data Center, Beijing Institute of Genomics, Chinese Academy of Sciences, under accession numbers (access No. HRA003299 and HRA003300) that can be accessed at https://ngdc.cncb.ac.cn/gsa-human/.

Field-specific reporting

Please select the one below that is the best fit for your research. If you are not sure, read the appropriate sections before making your selection.

☒ Life sciences ☐ Behavioural & social sciences ☐ Ecological, evolutionary & environmental sciences

For a reference copy of the document with all sections, see nature.com/documents/nr-reporting-summary-flat.pdf

Life sciences study design

All studies must disclose on these points even when the disclosure is negative.

Sample size	Sample size for scRNA-seq was determined by the availability of public resource when we started our projects<br>No statistical tests were performed for sample size calculation.<br>The exact number of samples used for each figure is informed in each legend or manuscript description.<br>sample size of experiments other than scRNA-seq analysis is primarily determined by the availability of samples, if sufficient samples are available, at least 3 samples were included to conduct the statistical analysis
Data exclusions	All criteria for data exclusion were pre-established.<br>Cells with less than 200 detected genes were removed, as well as cells with more than 20% mitochondrial content to exclude the possible empty droplet and dead cell according to commonly used cutoff in previous reports. In order to avoid interference with the analysis caused by potential doublets, cells with detected genes above 6,000 or identified by scrublet were also eliminated.
Replication	mIF staining of CD3e,CD11c,CD86, SPP1,CD44,CD31,CD68 was conducted in 9 patients tissue slices and replicated for 2 times each. no additional attempts were conducted due to the limited resouce of the clinical samples.
Randomization	All samples were randomly selected to conduct experiments only dependent on the availability of samples without specific selection
Blinding	There was no specific blinding grouping applied in this research

Reporting for specific materials, systems and methods

We require information from authors about some types of materials, experimental systems and methods used in many studies. Here, indicate whether each material, system or method listed is relevant to your study. If you are not sure if a list item applies to your research, read the appropriate section before selecting a response.

Materials & experimental systems

n/a	Involved in the study
☐	☒ Antibodies
☒	☐ Eukaryotic cell lines
☒	☐ Palaeontology and archaeology
☒	☐ Animals and other organisms
☐	☒ Human research participants
☐	☒ Clinical data
☒	☐ Dual use research of concern

Methods

n/a	Involved in the study
☒	☐ ChIP-seq
☒	☐ Flow cytometry
☒	☐ MRI-based neuroimaging

Antibodies

Antibodies used

Antibody used for mIF included:
CD3(CD3ε (D7A6E™) XP® Rabbit mAb #85061, CST, 1:100),
CD11c (Anti-CD11c Recombinant Rabbit Monoclonal Antibody [SI19-06], Huabio, 1:100),
CD86(Anti-CD86 antibody [EP1158-37] (ab269587), Rabbit monoclonal to CD86, Abcam,1:200),
SPP1(Recombinant Anti-Osteopontin antibody [EPR21139-316] (ab214050), Rabbit monoclonal to Osteopontin, Abcam, 1:2000),
CD44(Recombinant Anti-CD44 antibody [EPR18668] (ab189524), Rabbit monoclonal to CD44, Abcam,1:2000),
CD68(Recombinant Anti-CD68 antibody [EPR20545] (ab213363), Rabbit monoclonal to CD68,1:4000),
CD31(Recombinant Anti-CD31 antibody [EPR17259] (ab182981), Rabbit monoclonal to CD31, Abcam,1:2000),

Validation

CD3(CD3ε (D7A6E™) XP® Rabbit mAb #85061, CST, 1:100), Swiss Database?https://www.uniprot.org/uniprot/P07766, citation: Grohmann M, Wiede F, Dodd GT, et al. Obesity Drives STAT-1-Dependent NASH and STAT-3-Dependent HCC. Cell. 2018;175(5):1289-1306.e20. The antibody was validated by the company using IHC and WB. Please refer to the manufacturer's descriptions: https://www.cellsignal.cn/products/primary-antibodies/cd3e-d7a6e-xp-rabbit-mab/85061

CD11c (Anti-CD11c Recombinant Rabbit Monoclonal Antibody [SI19-06], Huabio, 1:100). Swiss Database: https://www.uniprot.org/uniprot/P20702. The antibody was validated by the company using IHC. Please refer to the manufacturer's descriptions: http://www.huabio.cn/product/CD11c-antibody-ET1606-19

CD86(Anti-CD86 antibody [EP1158-37] (ab269587), Rabbit monoclonal to CD86, Abcam,1:200), Swiss Database: https://www.uniprot.org/uniprot/P42081,citation: Victora GD, Dominguez-Sola D, Holmes AB, Deroubaix S, Dalla-Favera R, Nussenzweig MC. Identification of human germinal center light and dark zone cells and their relationship to human B-cell lymphomas Blood. 2012;120(11):2240-2248. The antibody was validated by the company using
CD86 knock out cell lines. Please refer to the manufacturer's descriptions: https://www.abcam.com/cd86-antibody-ep1158-37-ab269587.html

SPP1(Recombinant Anti-Osteopontin antibody [EPR21139-316] (ab214050), Rabbit monoclonal to Osteopontin, Abcam, 1:2000), Swiss Database: https://www.uniprot.org/uniprot/P42081citation: Lorena D, Darby IA, Gadeau AP, et al. Osteopontin expression in normal and fibrotic liver. altered liver healing in osteopontin-deficient mice. J Hepatol. 2006;44(2):383-390. The antibody was validated by company using IHC and WB, please refer to the manufacturer's descriptions: https://www.abcam.com/osteopontin-antibody-epr21139-316-ab214050.html

CD44(Recombinant Anti-CD44 antibody [EPR18668] (ab189524), Rabbit monoclonal to CD44, Abcam,1:2000), Swiss Database: https://www.uniprot.org/uniprot/P16070, citation: Park SY, Lee HE, Li H, Shipitsin M, Gelman R, Polyak K. Heterogeneity for stem cell-related markers according to tumor subtype and histologic stage in breast cancer. Clin Cancer Res. 2010;16(3):876-887.
And the antibody was validated by the company using CD44 knock out cell lines. Please refer to the manufacturer's descriptions: https://www.abcam.com/cd44-antibody-epr18668-ab189524.html

CD68(Recombinant Anti-CD68 antibody [EPR20545] (ab213363), Rabbit monoclonal to CD68,1:4000),
Swiss Database: https://www.uniprot.org/uniprot/P34810citation: Hashimoto A, Sarker D, Reebye V, et al. Upregulation of C/EBPα Inhibits Suppressive Activity of Myeloid Cells and Potentiates Antitumor Response in Mice and Patients with Cancer. Clin Cancer Res. 2021;27(21):5961-5978. The antibody was validated by the company using
mIF and IHC. Please refer to the manufacturer's descriptions: https://www.abcam.com/cd44-antibody-epr18668-ab189524.html

CD31(Recombinant Anti-CD31 antibody [EPR17259] (ab182981), Rabbit monoclonal to CD31, Abcam,1:2000), Swiss Database: https://www.uniprot.org/uniprot/P16284, citation: Pusztaszeri MP, Seelentag W, Bosman FT. Immunohistochemical expression of endothelial markers CD31, CD34, von Willebrand factor, and Fli-1 in normal human tissues. J Histochem Cytochem. 2006;54(4):385-395. The antibody was validated by the company using
IHC. Please refer to the manufacturer's descriptions: https://www.abcam.com/cd31-antibody-epr17259-ab182981.html

Human research participants

Policy information about studies involving human research participants

Population characteristics

ID	Gender	Status	Age	Diagnosis	TNM Stage	analysis
ATC_p1	M	Dead	69	Anaplastic thyroid cancer	T4bN1bM0 IV	mIF
ATC_p2	F	Dead	78	Anaplastic thyroid cancer	T4bN1aM1 IV	mIF
ATC_p3	F	Dead	78	Anaplastic thyroid cancer	T4aN1aM0 IV	mIF

March 2021

CRC_p1	M	Alive	44	Colon cancer	T3N0M1 IV	mIF+spRNA-seq
CRC_p2	M	Dead	64	Rectal cancer	T3N1M0 III	mIF+spRNA-seq
CRC_p3	M	Dead	45	Rectal cancer	T4N2M1 IV	mIF+spRNA-seq
CRC_p4	M	Alive	64	Colon cancer	T2N0M1 IV	spRNA-seq
CRC_p5	M	Alive	65	Rectal cancer	T4N0M1 IV	spRNA-seq
CRC_p6	F	Alive	62	Rectal cancer	T4N1bM1 IV	spRNA-seq
CRC_p7	M	Alive	68	Rectal cancer	T4N2bM1 IV	spRNA-seq
STAD_p1	F	Alive	45	Gastric Cancer	T3N2M0 III	mIF
STAD_p2	M	Alive	58	Gastric Cancer	T3N1M0 II	mIF
STAD_p3	M	Alive	68	Gastric Cancer	T3N2M0 III	mIF
STAD_p4	F	Alive	71	Gastric Cancer	T4bN1M0 III	mIF

Recruitment

All histologically diagnosed gastric/colorectal/ATC patients at West China Hospital (Chengdu, China) between January 2010 and May 2020 were retrospectively included. Two experienced pathologists reviewed slides independently to confirm the diagnosis following WHO criteria. Totally FFPE samples from 4,3,3 patients with gastric cancer, colorectal, and anaplastic thyroid cancer were used for mIF, respectively, while FFPE samples from 7 patients with colorectal cancer were used for spatial transcriptome.

Ethics oversight

clinical samples were used for mIF and spatial transcriptome analysis in this study. This study was approved by Ethics Committee of West China Hospital, Sichuan University (gastric cancer [2014, No. 215], colorectal cancer [2019, No. 338], and anaplastic thyroid cancer [2019, No. 592]), and informed consent was obtained from patients or their guardians, as appropriate. Sample collection was performed in compliance with Chinese laws and the Declaration of Helsinki.

Note that full information on the approval of the study protocol must also be provided in the manuscript.

Clinical data

Policy information about clinical studies

All manuscripts should comply with the ICMJE guidelines for publication of clinical research and a completed CONSORT checklist must be included with all submissions.

Clinical trial registration: N/A

Study protocol: N/A

Data collection: N/A

Outcomes: N/A
